# Disaggregating Digital Competence: Social Activity Participation and Depressive Symptoms Among Korean Older Adults

**DOI:** 10.3390/bs16101742

**Published:** 2026-09-24

**Authors:** Young-Mi Jung, NakHyeok Choi

**Affiliations:** 1 Department of Public Administration, Graduate School, Gachon University, Seongnam-si 13120, Republic of Korea; jj1885@korea.kr; 2 Department of Public Administration, Gachon University, Seongnam-si 13120, Republic of Korea

**Keywords:** digital competence, digital literacy, digital divide, depressive symptoms, older adults, social participation, mediation, educational inequality, National Survey of Older Koreans

## Abstract

Digital competence is commonly measured as a single summed score in research on late-life mental health, and a recent analysis of the same national survey data used here reported no adjusted association with depressive symptoms. This study aimed to describe how two domains of digital device competence relate to depressive symptoms among Korean older adults, and to test whether social activity participation accounts for part of these relations. Using the 2023 National Survey of Older Koreans (N = 9951), principal component analysis, supported by categorical confirmatory factor analysis and a split-sample check, yielded two domains: procedurally demanding life-service competence and everyday communication and media-use competence. When both domains were entered simultaneously, each was negatively associated with depressive symptoms, and bootstrap analyses indicated significant indirect associations through social activity participation for both. A 10-item summed score of the same items also showed significant direct and indirect associations as well, and first-stage and direct coefficients did not differ significantly between domains in the primary models, so disaggregation did not recover a relation that aggregation had concealed. Direct-path differences between domains and exploratory education moderation depended on model specification. Because the data are cross-sectional, the findings describe associations rather than effects.

## 1. Introduction

The Republic of Korea became a super-aged society in December 2024; as of 2025, approximately 10.51 million people aged 65 and over comprised 20.3% of the population (Statistics Korea, 2025). Against this demographic backdrop, late-life depression remains an important public health concern. The prevalence of depressive symptoms among Korean older adults declined modestly from 13.5% in 2020 to 11.3% in 2023, but more than one in ten older adults still experiences clinically relevant depressive symptoms, and the rate among those living alone (16.1%) is more than twice that among older couple households (7.8%) (E. Kang et al., 2023). Older adults living alone accounted for 32.8% of the older population in 2023. The number of people in this elevated-risk group continues to grow.

Social isolation is an established determinant of late-life depression, and the rapid digitization of everyday transactions—including restaurant ordering, banking, hospital registration, and public administration—has created an additional pathway to social isolation. Kiosk-based self-service can impose psychological costs on older adults with limited digital competence, who may experience the effort required as unpaid shadow work (Ji & Koh, 2023). Research on the digital divide has therefore shifted attention from binary physical access toward graded differences in material access and usage (van Deursen & van Dijk, 2019). This shift makes the content of digital use, rather than its mere presence, the analytically relevant quantity; Section 2 reviews these studies in detail.

A substantial body of evidence links digital engagement to mental health in later life. Internet exclusion is associated with more depressive symptoms across five international aging cohorts (Yan et al., 2024), while meta-analytic evidence links internet use to lower loneliness, with effect sizes varying systematically by type of use (Cao et al., 2025). Korean studies report similar associations for digital technology use and depressive symptoms (M.-A. Lee et al., 2021), latent digital literacy profiles associated with different levels of depressive-symptom risk (Shin et al., 2025), digitally mediated pathways to social participation (H. Lee et al., 2025), lower social isolation among those with greater device competence and longer usage time (S. Park et al., 2025), and a buffering role for social capital (Jeong & Bae, 2025).

Two limitations constrain what this literature can establish. The first concerns measurement. Digital competence is typically operationalized as a single summed count of functions performed, despite strong conceptual and psychometric grounds for treating digital skills as multidimensional. The validated Internet Skills Scale distinguishes operational, information-navigation, social, creative, and mobile skills (van Deursen et al., 2016). Similarly, a recently validated digital literacy scale developed specifically for older adults recovers four factors—basic technology, communication, problem-solving, and security literacy—with communication literacy clearly separated from the other domains (Yu et al., 2025). A scoping review of 42 quantitative studies of Korean older adults identified 25 distinct digital literacy measures, only three of which were validated instruments, and concluded that estimates remain inconclusive partly because of this measurement heterogeneity (H. Kang et al., 2023). Where studies distinguish domains of use, the distinction appears consequential: among Korean older adults, instrumental internet use (information and services) and interpersonal communication use show markedly different associations with both self-rated health and depressive symptoms (G.-S. Jeon & Choi, 2024). Procedurally demanding transactional tasks are also psychologically distinct from familiar communicative tasks. For example, technology anxiety in the use of digital public services operates almost entirely through perceived usefulness and self-efficacy rather than directly (An et al., 2024), while kiosk can elicit time pressure, negative emotion, and avoidance among older users (J. Nam et al., 2023).

The second limitation concerns mechanism: the direct association may not be the pathway through which digital competence matters most. In the most directly relevant precedent, M.-G. Kim et al. (2025) analyzed the same 2023 National Survey of Older Koreans used here, with the same analytic sample of 9951 respondents and the same geriatric depression screening instrument. They found that greater digital literacy was associated with fewer depressive symptoms in unadjusted analysis, but the association became non-significant after covariate adjustment, whereas associations with self-rated health and life satisfaction remained robust. They concluded that digital literacy could serve as “a potential enabler, but not a direct determinant” of mental health and suggested that competence may not buffer against depression unless it is embedded in socially meaningful contexts. This conclusion points directly to a mediated pathway that their design did not formally estimate. Y.-E. Park (2026) subsequently used the same 2023 survey to show that social participation partially mediates the association between a single summed digital-literacy score and depressive symptoms. What remains untested is whether this mediated pathway—and the accompanying direct association—differs across qualitatively distinct domains of digital competence. Put differently, when digital competence is disaggregated, does each dimension relate to depressive symptoms directly, or mainly through the social activity it enables?

The present study takes up both problems. The Korean studies closest to it—Roh (2024) on the 2020 wave, S. Jeon (2025) on the 2023 wave but with the sample restricted to older adults living alone, and Y.-E. Park (2026) on that wave in full—all operationalize digital literacy as a single summed score, so none of them can say whether procedurally demanding transactional tasks and familiar communicative and media tasks stand in the same relation to mental health, or whether the mediated pathway differs between them. This study aimed to describe how two domains of digital device competence relate to depressive symptoms among Korean older adults, and to test whether social activity participation accounts for part of those relations. We disaggregate digital device competence into two sub-dimensions—life-service competence, comprising procedurally complex transactional tasks, and everyday communication and media-use competence, comprising relatively familiar and repetitive tasks performed on a personal device—and examine this structure empirically through principal component analysis. We then estimate, for each dimension simultaneously, the direct association with depressive symptoms and the indirect association operating through social activity participation, and test whether these pathways vary by age, education, and household composition. Because that comparison turns on the scoring strategy, we also estimate the same models using a single summed score of the identical items. This operationalization became possible only with the 2023 survey wave, in which kiosk ordering and video calling were newly added, expanding the digital-device battery to 13 items; analyses based on the 2020 and earlier waves could not construct the transactional dimension.

The study addresses three research questions.

RQ1. Do the two indirect associations differ in magnitude?

RQ2. Do the direct associations between each dimension of digital competence and depressive symptoms vary by age, education, and household composition?

RQ3. Does the first-stage path from digital competence to social activity participation, and hence the indirect association, vary by age, education, and household composition?

This article proceeds as follows. The next section reviews the studies on late-life depression, social isolation, and the digital divide, and sets out the analytical framework that links them. We then describe the data, measures, and analytic strategy, where the hypotheses are stated alongside the procedures used to test them, and present the results of the principal component, regression, and bootstrap analyses. The final sections discuss the findings in relation to prior research and outline implications, limitations, and directions for future research.

## 2. Theoretical Background

### 2.1. Depression in Later Life: Concept and Determinants

Depression in later life is understood not as a simple mood disturbance but as the outcome of interacting physical, psychological, and social processes of aging. Old age concentrates cumulative experiences of loss—physical decline, retirement, and the contraction of relationships—that predispose older adults to depressive symptoms. Clinically, late-life depression often presents with diffuse somatic complaints, insomnia, and cognitive slowing rather than typical depressed mood, which can lead to confusion with dementia; social isolation and chronic disease act as principal contributing conditions.

For measurement, the Geriatric Depression Scale (GDS; Yesavage et al., 1982), whose yes/no format minimizes cognitive burden, and its short-form Korean validation (SGDS-K; Kee, 1996) are the most widely used instruments. The present study adopts the SGDS-K, whose validity and reliability have been documented extensively in Korean gerontological research.

### 2.2. Social Isolation, Network Contraction, and Mental Health in Old Age

Social isolation denotes a state of disconnection from social networks with insufficient interaction, and it is a potent risk factor for morbidity and mortality whose consequences are most profound among older adults (Cacioppo & Hawkley, 2003). Retirement, bereavement, illness, and reduced mobility make such disconnection especially likely in later life. In Korea, the weakening of family-centered networks and the spread of nuclear households have intensified isolation among older adults, with adverse consequences for emotional stability and mental health. Social isolation and loneliness are established risk factors for late-life depression and cognitive decline, and Korean evidence shows that discrimination experiences and social isolation are associated with higher odds of depression (Ko & Kwak, 2021).

Social network and social support theory provides the principal framework for this relationship. Berkman and Glass (2000) proposed a conceptual model in which social networks and support influence health through behavioral and psychobiological pathways, including social support, social influence, social engagement, and access to resources. In Korea, social networks mediate the association between older adults’ digital capability and depression (Kwon, 2022); perceived community environment moderates the association between digital utilization and social isolation (Y. Lee & Kim, 2025); both objective and subjective isolation are associated with depression, with coping flexibility attenuating the contribution of subjective isolation (G. Park, 2023); and during the COVID-19 period, social isolation was associated with lower life satisfaction through negative affect and self-esteem, with its adverse contribution larger among older adults living alone (Jung, 2024). These findings speak directly to the premise of the present study: social isolation is not only a core correlate of late-life depression but can combine with the digital divide to produce new forms of social exclusion. Social activity participation, the mediator examined in this study, is treated as a behavioral indicator that may facilitate social connectedness; it is not a direct measure of loneliness, perceived support, or social isolation.

### 2.3. Digital Divide and Digital Exclusion Theory

The digital divide refers to social inequality in access to and capability with information and communication technologies (ICT), encompassing not merely connectivity but differences in usage skill, digital literacy, and information competence. M. Kim and Kim (2002) proposed a three-dimensional model of access, usage, and acceptance, arguing that divides extend beyond device diffusion to capability and attitudes, and van Dijk (2005) conceptualized sequential divides in physical access, skills, and outcomes through which ICT can reproduce inequality in opportunities and resources. Older adults are among the groups most disadvantaged in both access to and use of digital devices, and digital exclusion carries over into quality of life, economic activity, and social participation (H. S. Kim & Shim, 2020). Korean survey data likewise show that within the older population the use of online public services rises steeply with household income—from 7.3% in households under 1 million won per month to 38.8% in those at 4 million won or more—even though access to devices and connectivity is near-universal, indicating that expanding infrastructure alone cannot close the divide (National Information Society Agency, 2025).

Digital exclusion designates the resulting exclusion of particular groups from socioeconomic opportunity (Warschauer, 2003). It extends beyond technical access to exclusion from social relationships, economic opportunity, and cultural participation; among older adults it feeds social isolation, information inequality, and intergenerational disconnection, undermining mental health and life satisfaction. Qualitative evidence documents older adults who remain marginalized within the older population itself as everyday services move online (S. Nam et al., 2022), and usage capability has been identified as a pivotal variable that can either mitigate or amplify social inequality (van Deursen & van Dijk, 2014). Helsper and Eynon (2010) further showed that generation is only one predictor of advanced internet use, alongside breadth of use, experience, gender, and education, which in some cases matter more—implying that the digital divide in later life is not a fixed generational trait but a domain improvable through education and support.

### 2.4. Digital Use Experience and the Competence–Social Activity–Depression Pathway

Studies of everyday digital device use converge on the finding that older adults’ digital competence matters for life satisfaction and mental health. Attitudes toward device use are associated with higher life satisfaction, an association mediated by the performance actually achieved with the device (S. K. Kim et al., 2021); the level of digital informatization is associated with life satisfaction (H. Lee & Won, 2024); and digital helpers mediate the association between device use performance and life satisfaction (Seo & Jung, 2024). Conversely, qualitative accounts describe heterogeneous experiences of marginalization within the older population as services move online (S. Nam et al., 2022), and technostress in kiosk use is associated with greater conflict over use among older adults (Ji & Koh, 2023).

Digital competence—the ability to search, process, and share information and to participate in socioeconomic activity through ICT (Ferrari, 2013)—extends beyond technical skill to literacy, problem-solving, and online collaboration. Among older Americans, internet use has been associated with lower depression, with propensity-score analyses estimating a 20% to 28% reduction in the likelihood of depression classification (Cotten et al., 2012). Multidimensional internet use is associated with lower depression partly through greater social participation (Du et al., 2023). Korean evidence shows that informatization education strengthens digital competence and everyday quality of life, although gains in smart-device competence and in economic outcomes are more limited (Y.-D. Kim et al., 2017); that lower device competence accompanies higher depression, moderated by geographic access to digital education services (Baek & Park, 2025); that the inverse association between digital literacy and depression is partially mediated by social activity, with the indirect association stronger among the old-old (Roh, 2024); that among Korean older men the association between internet use and depression varied with proxies for social isolation, with benefits concentrated among those in poor health or not employed (Jun & Kim, 2016); and that digital literacy predicts both satisfaction with social activity and participation in educational, fellowship, and religious activities, whereas subjective age perception predicts satisfaction only (H. S. Kim & Shim, 2020). That device competence and usage time—rather than mere ownership—predict lower isolation (S. Park et al., 2025) and that the isolation-reducing role of usage varies with perceived community environment (Y. Lee & Kim, 2025) indicate that the relationship between digital use and isolation requires a multidimensional approach attentive to the content and context of use.

### 2.5. Differences by Age, Education, and Household Composition

Age, education, and household composition are key background variables shaping digital literacy, social activity participation, and mental health. Digital literacy and social participation decline with age, and education bears directly on usage capability and participation, with more-educated groups more proficient with devices (E. Kang et al., 2023). Qualitative evidence documents substantial heterogeneity within the older population in how digital exclusion is experienced (S. Nam et al., 2022), while participation in informatization education strengthens digital competence and everyday quality of life, including interpersonal contact and leisure (Y.-D. Kim et al., 2017). International evidence likewise identifies age and education as core factors deepening the divide (van Deursen & van Dijk, 2014), and the finding that breadth of use, experience, and education can matter more than generation for advanced internet use (Helsper & Eynon, 2010) supports tailored interventions that take educational background into account. Differentiated digital education and support for older adults living alone and for less-educated, low-income groups may therefore contribute to social integration and the prevention of depression.

### 2.6. Analytical Framework

The analytical framework of this study integrates the studies on late-life depression, social isolation, the digital divide and digital exclusion, and digital use and social activity. Social network and support theory explains how network contraction and insufficient support undermine mental health in later life (Berkman & Glass, 2000); digital divide theory emphasizes that gaps in ICT access, usage, and outcomes constitute structural inequality arising differentially by age, education, and household composition (van Dijk, 2005; M. Kim & Kim, 2002); and digital exclusion theory shows that these gaps can culminate in social exclusion (Warschauer, 2003; S. Nam et al., 2022). Empirical findings that everyday digital competence promotes social activity participation and, through it, mental health (Du et al., 2023; Roh, 2024) ground the digital competence → social activity → depression pathway specified here.

Two features of the study design follow from this framework. First, the mediating role of social activity participation is estimated for both competence dimensions simultaneously, so each coefficient represents the association of one dimension after adjustment for the other. Second, moderation is examined at two distinct points: the direct associations between each competence dimension and depressive symptoms (RQ2) and the first-stage path from digital competence to social activity participation, indexed by the index of moderated mediation (RQ3). Roh (2024), by contrast, tested age moderation of the second-stage path from social activity participation to depressive symptoms (Hayes, 2018). This design is informative even when moderation is absent: in that case, no evidence of heterogeneity is detected under the tested specification. The framework is summarized in Figure 1.

## 3. Materials and Methods

### 3.1. Data and Participants

This study used the raw data of the 2023 National Survey of Older Koreans (E. Kang et al., 2023), obtained from the Microdata Integrated Service of Statistics Korea (Ministry of Health and Welfare & Korea Institute for Health and Social Affairs, 2023). The survey is a statutory national survey conducted under Article 5 of the Welfare of Senior Citizens Act. It targets adults aged 65 and over nationwide and collects information on sociodemographic characteristics, health status, social activity, economic activity, and digital device use through trained-interviewer household visits.

The initial sample comprised 10,078 respondents. Of these, 127 were coded as “not applicable” on the core measurement variables of this study—the 15 depression items and the 13 digital device use items—primarily because of proxy response. These cases were removed by listwise deletion, yielding a final analytic sample of 9951. This sample size and exclusion procedure are identical to those reported by M.-G. Kim et al. (2025) for the same dataset, which facilitates direct comparison between the two sets of findings.

### 3.2. Measures

#### 3.2.1. Depressive Symptoms

Depressive symptoms were measured with the 15-item Korean version of the Short-Form Geriatric Depression Scale (SGDS-K) (Kee, 1996), the Korean-validated adaptation of the instrument originally developed by Yesavage et al. (1982) and subsequently developed and validated in Korean by Bae and Cho (2004). Each item uses a yes/no response format, which minimizes the cognitive burden associated with Likert-type scales in older respondents. Five positively worded items (satisfaction with current life, feeling refreshed, cheerfulness, pleasure in being alive, feeling energetic) were reverse-coded so that a “no” response scored 1 as indicating a depressive symptom; the remaining ten items scored 1 for a “yes” response. Item scores were summed to yield a total score ranging from 0 to 15, with higher scores indicating greater depressive symptomatology. Internal consistency in the present sample was acceptable (Cronbach’s α = 0.842). We analyzed the continuous sum score rather than dichotomizing at the conventional clinical threshold in order to retain information across the full range of symptom severity.

#### 3.2.2. Digital Device Competence

Digital device competence was measured using the survey’s 13-item digital device use battery, in which respondents indicated whether they were able to perform each function (able = 1, unable = 0). To examine the dimensional structure of these items, we conducted a principal component analysis, retaining a two-component solution with Kaiser-normalized Varimax rotation. Two components with eigenvalues exceeding 1 were extracted (eigenvalue_1_ = 5.80, eigenvalue_2_ = 1.77); the full rotated loading matrix for all 13 items is reported in the Supplementary Material, Table S1.

The first dimension, labeled **life-service competence**, comprised e-commerce, financial transactions, application installation, and kiosk ordering (all loadings ≥ 0.71; 4 items, range 0–4, α = 0.842). These tasks share high procedural complexity, requiring sequential accuracy, security or payment handling, and tolerance of time pressure in public settings.

The second dimension comprised receiving messages, sending messages, taking photographs, searching for information, video calling, and watching videos (all loadings ≥ 0.67; 6 items, range 0–6, α = 0.875). We label it everyday communication and media-use competence, abbreviated below as communication-media competence. The label is deliberately broad. Three of the six items are interpersonal, but taking photographs, searching for information, and watching videos are solitary content use rather than communication. What the six items share is not communicative intent but the manner of use: they are performed on a familiar personal device, are repetitive, involve no payment or security step, and carry no time pressure in a public setting. We therefore do not interpret this dimension as a measure of interpersonal communication ability.

Three items were excluded from the final measures. Listening to music cross-loaded on both components; playing games showed only a modest primary loading without a clear conceptual home; and social networking service use loaded closer to the life-service dimension in a manner that was not conceptually interpretable. Retaining these items reduced both interpretability and internal consistency. This two-factor structure corresponds to the instrumental-versus-interpersonal distinction previously drawn among Korean older adults (G.-S. Jeon & Choi, 2024) and to the separation of communication skills from instrumental skills reported in validated multidimensional digital literacy instruments (van Deursen et al., 2016; Yu et al., 2025). A confirmatory factor analysis with WLSMV estimation for categorical indicators favored the correlated two-factor model over a single-factor model (CFI = 0.998 vs. 0.996; TLI = 0.998 vs. 0.995; RMSEA = 0.038 vs. 0.057; SRMR = 0.034 vs. 0.069; scaled Δχ^2^(1) = 303.7, *p* < 0.001), with standardized loadings of 0.90–0.94 for life-service and 0.84–0.99 for communication-media items and a factor correlation of 0.878 (see Supplementary Material, Table S5).

#### 3.2.3. Social Activity Participation

We measured social activity participation as the sum of participation in hobby or interest groups and in friendship or fellowship organizations (participating = 1, not participating = 0), producing a score ranging from 0 to 2, with higher scores indicating more active participation. This operationalization captures organized and formal group participation, which in prior chain-mediation work carried a larger indirect pathway to depressive symptoms than a friend-network measure did (Liu et al., 2024). This measure is a behavioral indicator of organized group participation; it does not directly measure loneliness, perceived support, or social isolation. It also excludes informal sociability—contact with family and relatives, and interaction with neighbors and friends—which the survey records separately.

#### 3.2.4. Covariates and Moderators

We dichotomized age into young-old (65–74 years = 0) and old-old (75 years and over = 1) and recoded education into three categories: no formal schooling (1), elementary school (2), and middle school or above (3). This operationalization matches that used in prior work on the same dataset (S. Jeon, 2025) and reflects the limited case count in higher educational categories, with only 6.3% of respondents reporting an associate degree or above. We dichotomized household composition into living alone (1) and not living alone (0); including older couple households, co-residence with children, and other household types). Sex was coded female = 1 and male = 0 and entered as a covariate.

### 3.3. Analytic Strategy

Primary analyses—descriptive statistics, hierarchical regression, and all bootstrap mediation and moderation analyses—were conducted in Stata/SE 16.1 (StataCorp, College Station, TX, USA). Categorical confirmatory factor analyses (WLSMV estimator) and the tetrachoric parallel analysis were conducted in R 4.6.1 using the lavaan (0.7–2) and psych packages. Nested model comparisons used the scaled chi-square difference test (Satorra, 2000). Selected regression and bootstrap estimates were independently cross-checked in Python 3.12 (pandas, statsmodels).

The analytical framework set out in Section 2.6 implies two hypotheses, stated here alongside the procedures used to test them.

**H1.**  *Life-service competence (H1a) and communication-media competence (H1b) are each independently and negatively associated with depressive symptoms after mutual adjustment. Both dimensions are entered simultaneously in the hierarchical regressions described below, and H1 is evaluated against the coefficients of the total-effect and direct-effect models*.

**H2.**  *Each competence dimension will show a negative indirect association with depressive symptoms through social activity participation (H2a, H2b). Each indirect association is estimated as a product of coefficients and tested with percentile 95% confidence intervals from 5000 bootstrap resamples*.

We estimated the direct associations of the two digital competence dimensions with depressive symptoms and with social activity participation using hierarchical regression. We assessed mediation with the product-of-coefficients approach and tested it with 5000 bootstrap resamples (Hayes, 2018), from which percentile 95% confidence intervals for indirect effects were derived. We tested the difference between the two indirect effects directly by bootstrapping the difference distribution.

We tested moderation of the direct paths by entering interaction terms between each digital competence dimension and each moderator (age, education, household composition). We tested moderated mediation by specifying moderation of the first-stage (a) path from digital competence to social activity participation, and by bootstrapping the index of moderated mediation for each first-stage interaction. Continuous variables were mean-centered prior to forming interaction terms in order to reduce multicollinearity. Statistical significance was evaluated at α = 0.05. Both digital competence dimensions were entered simultaneously in all models, so that each coefficient represents the association of that dimension net of the other. We added four sets of sensitivity analyses. First, because the two competence scales differ in length (0–4 vs. 0–6), the indirect associations were re-estimated in fully standardized form and their difference was bootstrapped. Second, all models were re-estimated with an extended covariate set—marital status, employment, self-rated health, number of chronic conditions, ADL and IADL limitations, cognitive function score, and fall experience. Third, models were re-estimated applying the survey’s standardized post-stratification weight with cluster-robust standard errors and cluster resampling at the enumeration-district level (977 districts). Fourth, the dimensional structure was probed with parallel analysis of the tetrachoric correlation matrix and a random split-sample check, in which principal component analysis in one random half was followed by confirmatory factor analysis in the other. We also re-estimated the primary models using a single summed score of the ten retained items. Because that score is the sum of the two subscales, a model using it is the disaggregated model with a common coefficient imposed on the two dimensions, and we tested that equality constraint directly.

Figure 2 summarizes the analytical workflow, from assessment of dimensional structure to the primary models, pathway tests, and sensitivity analyses.

## 4. Results

### 4.1. Sample Characteristics

Women comprised 61.6% of the sample and men 38.4%. Young-old respondents (65–74 years) accounted for 57.4% and old-old respondents (75 years and over) for 42.6%. Respondents living alone comprised 34.4% of the sample, closely matching the 32.8% figure reported for the older population in the 2023 survey report (E. Kang et al., 2023) and indicating that the analytic sample adequately reflects the household composition of the target population. Full sample characteristics appear in Table 1.

### 4.2. Item-Level Digital Competence

The proportion of respondents able to perform each life-service task ranged from 10.8% to 17.9%, markedly lower than the range for communication-media tasks (41.0% to 81.6%). Item-level distributions appear in Table 2.

This asymmetry is consistent with Korean older adults being comparatively familiar with basic communicative and media functions such as messaging and photography, while experiencing substantial difficulty with problem-solving service tasks such as e-commerce, financial transactions, and kiosk ordering. The magnitude of the gap—a more than twofold difference in prevalence between the least common communication-media task and the most common life-service task—is a descriptive difference in diffusion; the evidence bearing on whether the two form distinct dimensions is reported in Section 3.2.2 and Section 4.6.

### 4.3. Descriptive Statistics and Correlations

Life-service competence (M = 0.559, SD = 1.139) was substantially lower relative to its maximum than communication-media competence (M = 3.306, SD = 2.227), confirming a pronounced difference in the diffusion of the two types of digital use. Skewness (|value| < 3) and kurtosis (|value| < 10) for all continuous variables fell within conventional bounds for normality, and all reliability coefficients exceeded 0.70.

Both life-service competence (r = −0.206) and communication-media competence (r = −0.266) were significantly negatively correlated with depressive symptoms. The correlation between the two digital competence dimensions (r = 0.504) was well below the threshold conventionally taken to indicate problematic multicollinearity (0.80), supporting their simultaneous entry as distinct predictors. Social activity participation was negatively correlated with depressive symptoms (r = −0.265) and positively correlated with both digital competence dimensions (r = 0.364 and r = 0.468, respectively), consistent with the specified mediation structure. Correlations appear in Table 3.

### 4.4. Direct Associations and Mediation by Social Activity Participation

Table 4 presents the hierarchical regression models. Model 1 regresses social activity participation on the two digital competence dimensions and covariates; Model 2 estimates the total association with depressive symptoms without the mediator; and Model 3 adds the mediator to estimate direct associations.

We start with the results bearing on the two hypotheses. In Model 1, both life-service competence (β = 0.157, *p* < 0.001) and communication-media competence (β = 0.313, *p* < 0.001) were positively associated with social activity participation, with the model accounting for 25.1% of variance; the standardized coefficient for communication-media competence was approximately twice as large. In Model 2, both dimensions showed significant negative total associations with depressive symptoms (β = −0.088 and β = −0.159, respectively, both *p* < 0.001), supporting H1a and H1b. In Model 3, social activity participation was negatively associated with depressive symptoms (β = −0.161, *p* < 0.001), and both digital competence dimensions retained significant negative direct associations (β = −0.063 and β = −0.109, both *p* < 0.001).

We note that these associations, while highly significant, are modest in magnitude: the full model accounts for 10.7% of variance in depressive symptoms, and the addition of the mediator increased explained variance by 1.9 percentage points over the total-effect model. Given the sample size, statistical significance should not be read as an indicator of practical magnitude, and we interpret the pattern of coefficients rather than their significance alone throughout.

We turn next to the indirect associations. Bootstrap results appear in Table 5. The indirect association for life-service competence was −0.072 (95% CI [−0.086, −0.059]) and for communication-media competence −0.073 (95% CI [−0.085, −0.063]); neither confidence interval included zero, supporting H2a and H2b. Because both direct associations remained significant in Model 3, the pattern is consistent with partial mediation for both dimensions, although cross-sectional data cannot establish causal mediation. Addressing RQ1, the difference between the two indirect associations on the raw per-task scale was 0.001; this fails to detect a difference but does not establish equivalence, and the two competence scales differ in length (0–4 vs. 0–6). In fully standardized terms, the indirect association was −0.025 for life-service and −0.051 for communication-media competence, and their difference excluded zero. In standardized terms the indirect association was therefore approximately twice as large for communication-media competence, reflecting its stronger first-stage association with social activity participation in standardized units (β = 0.313 vs. 0.157), which follows from the wider diffusion of those tasks rather than from a larger per-task association.

Finally, we compared the two-dimensional scoring with a single summed score of the same ten items (Supplementary Material, Table S9). The summed score was positively associated with social activity participation (B = 0.084, *p* < 0.001) and negatively with depressive symptoms, both in total and directly, and its indirect association through social activity participation was significant. In the disaggregated model the two coefficients did not differ significantly, either for the first-stage path or for the direct path, so the equality constraint that a summed score imposes was not rejected in the primary specification. Disaggregation therefore did not recover an association that aggregation had concealed; it described how two groups of digital tasks related to social activity participation and depressive symptoms, without establishing that those relations differ.

### 4.5. Moderation and Moderated Mediation

Table 6 summarizes interaction terms for the direct paths. Age did not significantly moderate the direct association for either dimension. Education significantly moderated the direct association for communication-media competence (B = 0.085, *p* = 0.001). Because the interaction coefficient is positive, the magnitude of the negative association between communication-media competence and depressive symptoms diminishes as education rises—that is, the association is strongest among older adults with the least education. The corresponding interaction for life-service competence was not significant (B = −0.162, *p* = 0.142). Household composition did not moderate either direct association. Because education is a three-category variable, the linear interaction treats adjacent categories as equidistant and should be read as an exploratory ordinal-trend test. When education was instead dummy-coded (reference: no formal schooling), the four interaction terms were not jointly significant (F = 2.08, *p* = 0.080); the communication-media interactions were of similar size for elementary schooling (B = 0.125, 95% CI [−0.003, 0.252]) and middle school or above (B = 0.124, 95% CI [0.001, 0.247]), suggesting that the stronger inverse association is specific to older adults with no formal schooling rather than following a graded educational trend. The interaction block for education added ΔR^2^ = 0.0011 (f^2^ = 0.0012), below Cohen’s (1988) conventional threshold for a small effect (Supplementary Material, Table S3). Together with its attenuation under extended adjustment (Section 4.6), the education moderation should be regarded as exploratory.

Finally, regarding moderated mediation (RQ3), the index of moderated mediation was non-significant for every combination of dimension and moderator. For age, the indices were −0.030 (95% CI [−0.070, 0.009]) for life-service competence and 0.001 (95% CI [−0.010, 0.011]) for communication-media competence. For education, they were 0.0064 (95% CI [−0.027, 0.039]) and −.0015 (95% CI [−0.009, 0.006]). For household composition, they were 0.0036 (95% CI [−0.018, 0.026]) and −0.0022 (95% CI [−0.012, 0.008]). No evidence of moderated mediation was detected.

### 4.6. Sensitivity Analyses

Four sets of sensitivity analyses assessed robustness (Supplementary Material, Tables S2 and S4–S8). Weight- and cluster-adjusted estimation applying the survey weight with cluster-robust standard errors across 977 enumeration districts left every focal association intact (life-service B = −0.244, communication-media B = −0.112, social activity participation B = −1.008, all *p* < 0.001), and a cluster-resampled weighted bootstrap confirmed both indirect associations (life-service −0.089, 95% CI [−0.113, −0.065]; communication-media −0.083, 95% CI [−0.101, −0.067]). Under the extended covariate set (N = 9837), both indirect associations remained significant (life-service −0.031, 95% CI [−0.040, −0.022]; communication-media −0.028, 95% CI [−0.036, −0.021]) and the life-service direct association persisted (B = −0.081, *p* = 0.004), but the communication-media direct association (B = 0.012, *p* = 0.507) and its moderation by education (B = 0.006, *p* = 0.780) attenuated to non-significance. Because several extended covariates—self-rated health, functional limitations, and cognitive function—are plausibly on the causal pathway from digital competence to depressive symptoms or share causes with it, the extended models may over-control; we therefore treat the demographic-adjusted models as primary and the extended models as a conservative robustness bound. The standardized difference between the two indirect associations also persisted under extended adjustment (0.009, 95% CI [0.005, 0.013]). Finally, the dimensional structure itself was probed. Parallel analysis of the tetrachoric correlation matrix (principal-component criterion) favored a single dominant factor (first two eigenvalues 9.91 and 0.77), whereas the categorical confirmatory model favored two factors with a high factor correlation (Section 3.2.2). A random split-sample check addressed the circularity of deriving and confirming the structure in the same sample: principal component analysis in one random half (n = 4975) reproduced the two-component solution (eigenvalues 5.83 and 1.76, identical item assignment), and a confirmatory model in the other half (n = 4976) again favored the two-factor solution over one factor (CFI = 0.998 vs. 0.995; RMSEA = 0.040 vs. 0.062; SRMR = 0.034 vs. 0.068; scaled Δχ^2^(1) = 194.5, *p* < 0.001; factor correlation = 0.87). Taken together, the two dimensions are distinguishable but highly correlated subdomains. In the summed-score sensitivity analyses (Table S9), the direct association remained significant under weighted primary adjustment (B = −0.157, *p* < 0.001), but not under extended adjustment, either unweighted (B = −0.019, *p* = 0.142) or weighted (B = −0.015, *p* = 0.451). The two domain coefficients differed for the direct path, but not for the first-stage path, in these sensitivity specifications, while all summed-score indirect associations remained significant. The contrast with the primary specification indicates that evidence of differences between domains depends on model specification.

## 5. Discussion

### 5.1. Principal Findings

This study disaggregated digital device competence among Korean older adults into life-service and communication-media domains and estimated, for each, the direct association with depressive symptoms and the indirect association operating through social activity participation. Three findings stand out. First, both domains were independently and negatively associated with depressive symptoms after mutual adjustment, and both were positively associated with social activity participation. Second, both domains showed significant indirect associations through social activity participation, and a single summed score of the same items reproduced those associations; the first-stage and direct coefficients did not differ significantly between domains in the primary specification, so disaggregation did not by itself recover relations that a summed score had concealed. Third, the larger standardized indirect association for communication-media competence persisted under extended adjustment. In contrast, evidence of between-domain differences in the direct associations and education moderation depended on model specification and should be read as exploratory.

### 5.2. Reconciling the Present Findings with Prior Work on the Same Dataset

The most consequential comparison is with M.-G. Kim et al. (2025), who analyzed the identical dataset with an identical analytic sample and found that the association between digital literacy and depressive symptoms, significant in unadjusted analysis, became non-significant after covariate adjustment. They interpreted digital literacy as “a potential enabler, but not a direct determinant” of mental health and suggested that its benefits may require socially meaningful contexts to materialize.

Our findings are not in conflict with theirs; they provide evidence consistent with the indirect pathway suggested by that conclusion. Three differences in analytic approach bear on the divergence in the direct association. First, M.-G. Kim et al. (2025) treated digital literacy as a unidimensional count across eight smartphone functions, whereas we used a 13-item battery, so the item sets differ. Scoring strategy alone does not account for the divergence: a summed score of our ten retained items showed the same significant direct association as the disaggregated model (Section 4.4). Second, they modeled depression dichotomously at the clinical screening threshold, whereas we modeled symptom severity continuously. Third, they adjusted for a broader covariate set including health, functioning, and cognition; consistent with this, our own extended-covariate model reproduced their null for the communication-media direct association, although the life-service direct association remained significant (Section 4.6). Separately from the direct-association question, we estimated the indirect pathway that their design did not. The significant indirect associations we observe through social activity participation are precisely what an “enabler” account predicts: the results are consistent with an indirect association operating through social activity participation rather than with a direct effect of digital competence itself. Under extended adjustment, both indirect associations and the life-service direct association remained significant, whereas the communication-media direct association did not—what persists across adjustment strategies, for both dimensions, is the pathway through social participation.

It is also consistent with Y.-E. Park (2026), who reported partial mediation by social participation for a single summed digital-literacy score in this same dataset; the present analysis extends that finding by estimating the mediated pathway for each competence dimension separately. This reading is consistent with the broader literature. Multidimensional internet use is inversely associated with depression partly through social participation in large Chinese national samples (Du et al., 2023), with mediated proportions in the range of 12% to 14% (Wu et al., 2025); our indirect associations correspond to roughly 29% of the total association for life-service competence and 32% for communication-media competence, somewhat larger but of the same order. Chain-mediation work in which participation in organized volunteer activity carried a larger indirect pathway than a friend-network measure (Liu et al., 2024) supports our operationalization of the mediator as formal group participation. Korean evidence that intensity of social participation shows a dose–response relationship with depression across activity types (M.-G. Kim & Choi, 2024) further corroborates the mediator-to-outcome link, although behavioral-activation evidence cautions that such benefits are activity-type-specific: in one collaborative-care trial, implemented self-care and spiritual activities were associated with symptom improvement whereas social activities showed no significant association (Boczor et al., 2025).

### 5.3. The Value of Disaggregation

The two-dimensional solution is theoretically interpretable, replicated in a random split-sample check (Section 4.6), and consistent with prior instruments, although it remains a within-survey structure requiring independent validation. Validated multidimensional instruments consistently separate communicative from instrumental digital skills (van Deursen et al., 2016; Yu et al., 2025), and a recent four-factor digital literacy scale developed specifically for older adults reproduces this separation. Among Korean older adults specifically, G.-S. Jeon and Choi (2024) had already shown that instrumental internet use and interpersonal communication use carry different associations with self-rated health and depressive symptoms. What the present study adds is a domain-level description within a single nationally representative battery: both dimensions retain negative associations with depressive symptoms when entered simultaneously, and both operate partly through social activity participation. Because a summed score of the same items yielded the same pattern (Section 4.4), this should be read as a more detailed account of an association that aggregation also detects, not as the recovery of one that aggregation had concealed.

One divergence deserves comment. G.-S. Jeon and Choi (2024) found the instrumental dimension more strongly associated with depressive symptoms than the interpersonal dimension, whereas we found the reverse (β = −0.109 for communication-media versus −0.063 for life-service). The most plausible explanation lies in the distributions. In our sample, only 10.8% to 17.9% of respondents could perform each life-service task, compared with 41.0% to 81.6% for communication-media tasks. A predictor on which the overwhelming majority of the sample scores zero has restricted variance and correspondingly limited capacity to explain variation in the outcome. This is substantively meaningful rather than merely technical: at present levels of diffusion in the Korean older population, transactional digital competence is too rare to carry much population-level association with mental health, however consequential it may be for the minority who possess it. As kiosk-based and app-based service delivery continues to expand, this balance may shift.

### 5.4. Education and the Absence of Moderated Mediation

In the primary specification, communication-media competence was more strongly associated with lower depressive symptoms among the least educated. This exploratory pattern is consistent with qualitative work documenting heterogeneity within the older population (S. Nam et al., 2022) and with evidence that digital exclusion falls disproportionately on those with fewer socioeconomic resources (Yang et al., 2024). The life-service interaction was not significant, but this does not establish that moderation differs between the two domains. This moderation should nonetheless be interpreted cautiously: it did not survive the extended covariate adjustment described in Section 4.6, suggesting that part of the education gradient reflects correlated differences in health, functioning, and cognition. In the primary specification, the dummy-coded analysis suggested a contrast between older adults with no formal schooling and those with schooling, rather than a graded educational pattern (Section 4.5).

The uniform absence of moderated mediation warrants interpretation rather than dismissal. Prior Korean work reported a significant index of moderated mediation for age (−0.022, 95% CI [−0.031, −0.013]; Roh, 2024), which may appear to conflict with our null results. The two designs, however, locate moderation at different points in the pathway: Roh tested age moderation of the second-stage (b) path from social activity to depression, whereas we tested moderation of the first-stage (a) path from digital competence to social activity. Read together, the two sets of results suggest that age-related variation may be confined to the b path, although cross-study comparison cannot establish this, while no moderation of the first-stage (a) path from digital competence to social activity participation was detected for age, education, or household composition. Null results of this kind are informative: no heterogeneity in the association linking digital competence to social participation was detected under the tested specifications, and promoting participation may therefore be worth examining across the older population rather than only within targeted strata.

### 5.5. Implications

Two implications follow, both tentative. The education-specific pattern was small, did not survive extended adjustment, and rests on a single interaction term; it is therefore not a basis for differentiating training programs by educational background, although it is a reason to examine whether basic everyday device functions matter more for older adults with the fewest educational resources. For the population as a whole, because indirect associations through social activity participation were observed for both dimensions and proved robust across all sensitivity analyses, programs that deliberately connect newly acquired digital skills to actual participation in hobby groups, volunteer activity, and community organizations may be promising irrespective of which skills are taught. Digital literacy programs that end at skill acquisition leave this participation pathway unaddressed.

### 5.6. Limitations

Several limitations qualify these findings. First, the data are cross-sectional, so causal ordering cannot be established. The relationships among digital competence, social activity, and depressive symptoms are plausibly bidirectional—older adults with depressive symptoms may withdraw from both digital learning and social activity—and the reverse pathway has in fact been modeled in this literature, with offline social participation predicting digital literacy (J. Kim et al., 2024). Longitudinal designs are needed to adjudicate direction. The same applies to the association between age and digital competence: advancing age may both lower demonstrated competence and reduce the motivation to acquire it, and cross-sectional data cannot separate the two.

Second, both digital competence and depressive symptoms rely on self-report, introducing potential measurement error from subjective appraisal. Self-reported ability to perform a digital task is not equivalent to demonstrated performance. Relatedly, because all measures were drawn from a single survey of the same respondents, common method variance may have inflated the observed associations (Podsakoff et al., 2003). Three features of the design attenuate, though they cannot eliminate, this risk: the competence and participation items are factual behavioral reports rather than attitudinal ratings, the survey was administered by trained interviewers rather than self-completed, and the depression scale contains five reverse-coded items. Shared method variance also tends to attenuate rather than generate interaction effects (Siemsen et al., 2010), and is therefore unlikely to account for the moderation pattern observed here.

Third, the sub-dimensional structure was derived post hoc from the 13 available survey items, and principal component analysis was applied to binary indicators, which can distort the estimated structure. Two sensitivity analyses qualify the structure in opposite directions. A categorical confirmatory factor analysis (WLSMV) favored the correlated two-factor model over a one-factor model (scaled Δχ^2^(1) = 303.7, *p* < 0.001; RMSEA 0.038 vs. 0.057; SRMR 0.034 vs. 0.069), supporting the distinction; at the same time, parallel analysis of the tetrachoric correlation matrix (principal-component criterion) favored a single dominant factor (first two eigenvalues = 9.91 and 0.77), and the factor correlation in the confirmatory model was high (0.878), indicating a strong general competence factor underlying both dimensions (see Supplementary Material, Tables S2 and S5). A random split-sample check mitigated, though it cannot fully remove, the circularity of deriving and testing the structure within the same survey (Section 4.6). The two dimensions should therefore be interpreted as statistically separable but highly correlated sub-domains of a broader digital competence continuum—which nonetheless differ markedly in prevalence and in their observed moderation pattern—rather than as fully independent constructs. Validation against a purpose-built instrument is needed before the two-dimensional structure can be treated as established; we report it as empirically supported within this dataset and consistent with prior multidimensional instruments, not as a validated scale.

Fourth, the survey employs a complex sampling design. The primary analyses did not incorporate weights or clustering, but weight- and cluster-adjusted sensitivity analyses applying the standardized post-stratification weight with enumeration-district cluster-robust standard errors and cluster resampling reproduced all focal associations (Section 4.6). Primary estimates should nonetheless be read as sample-based associations, and because household income and region are not included in the public microdata file, residual confounding by economic resources and area characteristics remains possible.

Fifth, the mediator was measured narrowly, as participation in hobby groups and friendship organizations (range 0–2). It does not capture volunteering, religious activity, lifelong learning, or informal sociability such as contact with family and relatives and interaction with neighbors and friends. It therefore represents organized participation rather than social connectedness as a whole, and a fuller account of the participation pathway would require broader measurement.

Sixth, the analysis relied on regression and bootstrapping rather than structural equation modeling; overall model fit assessment and multi-group cross-validation remain for future work.

## 6. Conclusions

Digital competence among older adults may comprise distinguishable but highly correlated subdomains rather than a single capacity. Disaggregating a national digital-device battery into procedurally demanding life-service tasks and everyday communication and media-use tasks yielded two domains, each negatively associated with depressive symptoms and each positively associated with social activity participation, which accounted for part of both relations. A single summed score of the same ten items reproduced this pattern, and the first-stage and direct coefficients did not differ significantly between domains in the primary specification. Disaggregation therefore provided a more detailed description of how different groups of digital tasks related to the outcomes, rather than recovering relations that aggregation had concealed. Between-domain differences in direct associations and the education moderation of the communication-media direct association were sensitive to model specification and remain exploratory.

These findings are consistent with the reading of digital literacy as a potential enabler rather than a direct determinant of late-life mental health, and they identify the participation pathway as the element that persisted across every specification examined. Because the data are cross-sectional, the relations reported here are associations rather than effects. On this basis, programs that connect newly acquired digital skills to actual participation in hobby groups, volunteer activity, and community organizations are worth examining irrespective of which skills are taught; the present results do not support differentiating such programs by educational background.

## Figures and Tables

**Figure 1:**
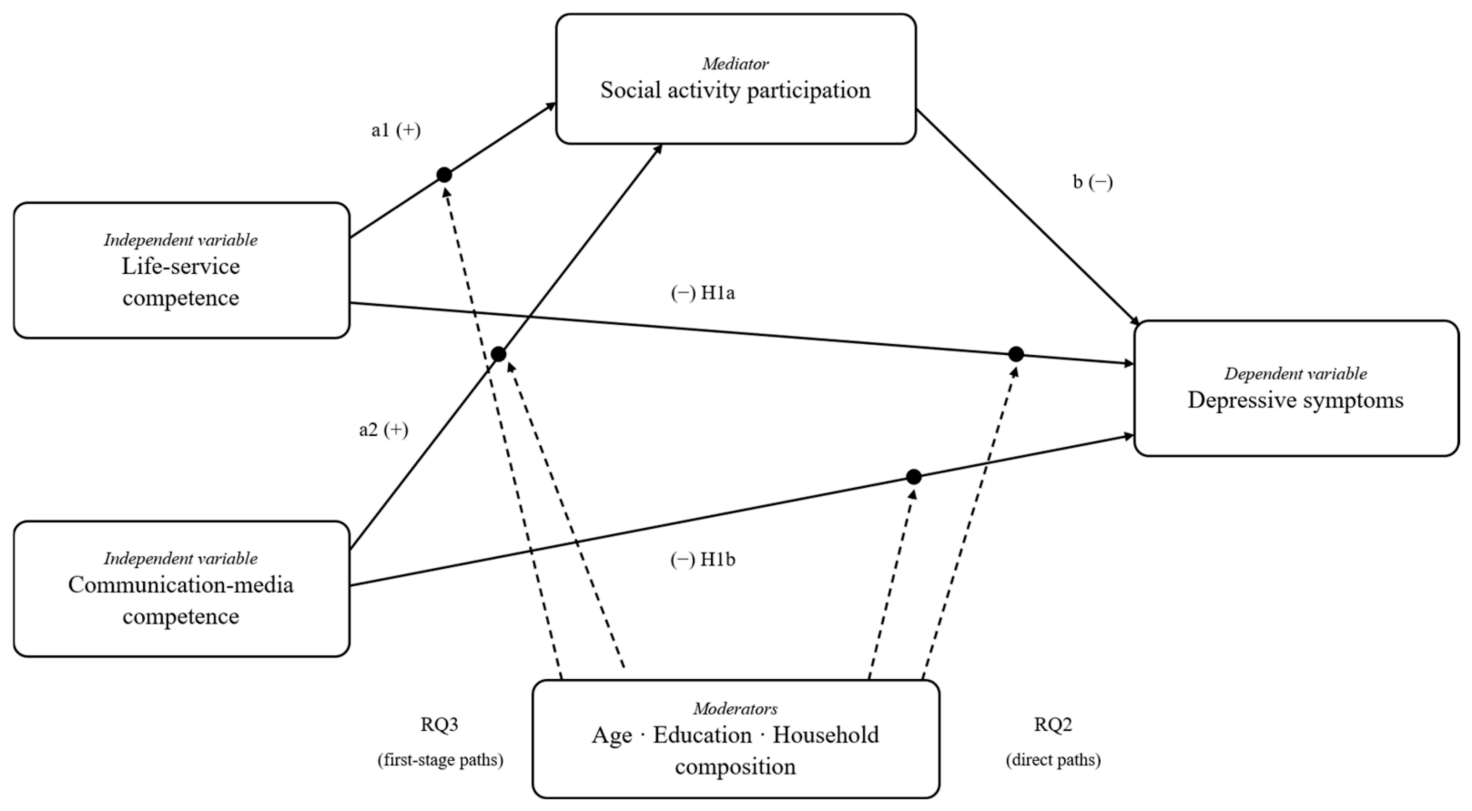
Conceptual model of the study. Solid arrows denote the first-stage paths a1 and a2 (digital competence → social activity participation), the second-stage path b (participation → depressive symptoms), and the direct associations H1a and H1b; the hypothesized indirect associations (H2a, H2b) correspond to a1 × b and a2 × b. Dashed arrows denote the moderation examined as research questions RQ2 (direct paths) and RQ3 (first-stage indirect paths).

**Figure 2:**
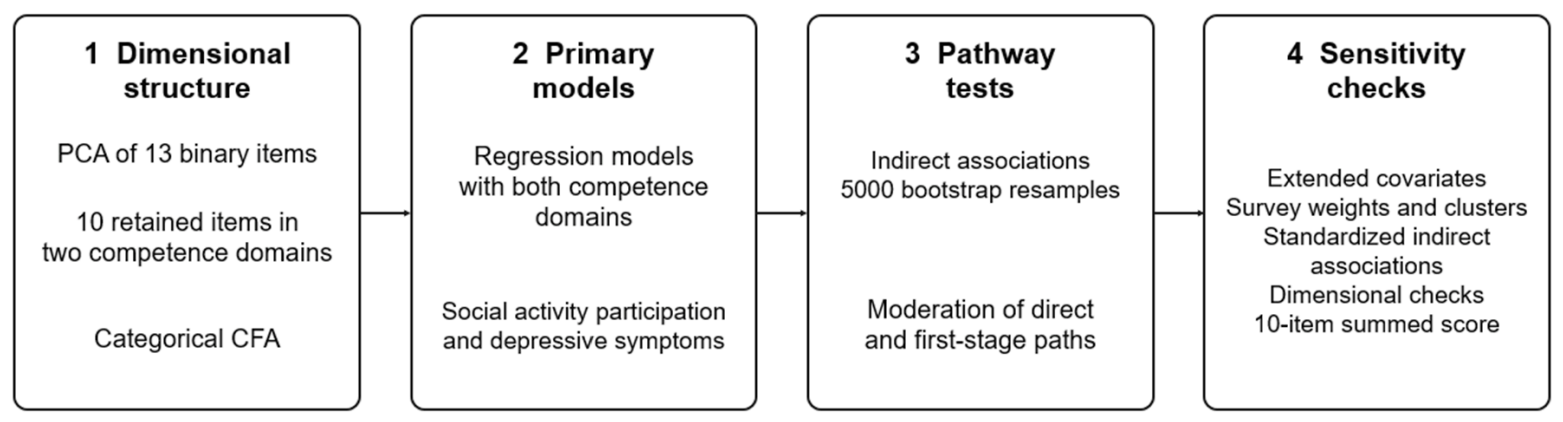
Overview of the analytical workflow.

**Table 1:** Sociodemographic characteristics of the sample.

Variable	Category	n	%
Sex	Male	3824	38.4
	Female	6127	61.6
Age group	Young-old (65–74)	5710	57.4
	Old-old (75+)	4241	42.6
Education	No formal schooling	1435	14.4
	Elementary school	2920	29.3
	Middle school	2114	21.2
	High school	2860	28.7
	Associate degree or above	622	6.3
Household type	Living alone	3423	34.4
	Older couple household	5419	54.5
	Co-residing with children	940	9.4
	Other	169	1.7
Total		9951	100.0

Note. Education was recoded into three categories for analysis: no formal schooling (1), elementary school (2), middle school or above (3).

**Table 2:** Item-level distribution of digital device competence and rotated component loadings.

Dimension	Item	Able (n)	Able (%)	Loading
Life-service	E-commerce	1074	10.8	**0.80**
	Financial transactions	1782	17.9	**0.76**
	Application installation	1182	11.9	0.80
	Kiosk ordering	1529	15.4	**0.72**
Communication-media	Receiving messages	8120	81.6	**0.77**
	Sending messages	7021	70.6	**0.84**
	Taking photographs	4895	49.2	**0.76**
	Searching for information	4626	46.5	**0.75**
	Video calling	4158	41.8	**0.67**
	Watching videos	4077	41.0	**0.67**

Note. Principal component analysis with varimax rotation; two components with eigenvalues > 1 were retained (5.80, 1.77). All life-service loadings ≥ 0.71; all communication-media loadings ≥ 0.67. Listening to music, playing games, and social networking service use were excluded (see Section 3.2.2).

**Table 3:** Descriptive statistics and correlations among study variables.

Variable	1	2	3	4	5	6
1. Depressive symptoms	—					
2. Life-service competence	−0.206 ***	—				
3. Communication-media competence	−0.266 ***	0.504 ***	—			
4. Social activity participation	−0.265 ***	0.364 ***	0.468 ***	—		
5. Age (old-old = 1)	0.173 ***	−0.342 ***	−0.506 ***	−0.317 ***	—	
6. Education	−0.211 ***	0.349 ***	0.574 ***	0.325 ***	−0.435 ***	—
7. Living alone (=1)	0.134 ***	−0.111 ***	−0.195 ***	−0.140 ***	0.142 ***	−0.230 ***
M	3.094	0.559	3.306	0.596	—	2.418
SD	3.235	1.139	2.227	0.600	—	0.729

Note. *** *p* < 0.001. Age (old-old = 1) and household type (living alone = 1) are dummy variables; their means and standard deviations are omitted. Education is the three-category recoded value.

**Table 4:** Hierarchical regression models for social activity participation and depressive symptoms.

Predictor	Model 1: Social Activity B (β)	Model 2: Depression, Total B (β)	Model 3: Depression, Direct B (β)
(Constant)	0.285 ***	4.488 ***	4.736 ***
Female (ref. male)	−0.048 *** (−0.039)	−0.093 (−0.014)	−0.134 * (−0.020)
Old-old (ref. young-old)	−0.102 *** (−0.084)	0.156 * (0.024)	0.067 (0.010)
Education	0.030 ** (0.036)	−0.282 *** (−0.064)	−0.256 *** (−0.058)
Living alone (ref. not alone)	−0.040 *** (−0.032)	0.533 *** (0.078)	0.498 *** (0.073)
Life-service competence	0.083 *** (0.157)	−0.251 *** (−0.088)	−0.179 *** (−0.063)
Communication-media competence	0.084 *** (0.313)	−0.231 *** (−0.159)	−0.158 *** (−0.109)
Social activity participation			−0.870 *** (−0.161)

Note. * *p* < 0.05, ** *p* < 0.01, *** *p* < 0.001. Model 1: R^2^ = 0.251, F(6, 9944) = 555.87 ***; Model 2: R^2^ = 0.088, F(6, 9944) = 159.49 ***; Model 3: R^2^ = 0.107, F(7, 9943) = 170.69 ***. β denotes fully standardized coefficients.

**Table 5:** Bootstrapped indirect associations via social activity participation.

Pathway	Indirect Estimate	95% CI
Life-service → social activity → depression	−0.072	[−0.086, −0.059]
Communication-media → social activity → depression	−0.073	[−0.085, −0.063]
Difference (life-service − communication-media)	0.001	[−0.011, 0.014]
Life-service → social activity → depression (fully standardized)	−0.025	[−0.030, −0.021]
Communication-media → social activity → depression (fully standardized)	−0.051	[−0.058, −0.043]
Difference, standardized (life-service − communication-media)	0.025	[0.019, 0.032]

**Table 6:** Moderation of direct associations with depressive symptoms.

Interaction Term	B	SE	*p*
Life-service × old-old	0.098	0.112	0.382
Communication-media × old-old	0.012	0.036	0.735
Life-service × education	−0.162	0.110	0.142
Communication-media × education	0.085	0.025	0.001
Life-service × living alone	0.009	0.072	0.900
Communication-media × living alone	0.047	0.034	0.172

## Data Availability

Restrictions apply to the availability of the microdata. The 2023 National Survey of Older Koreans microdata were obtained from the Microdata Integrated Service of Statistics Korea (https://mdis.kostat.go.kr; dataset DOI: https://doi.org/10.23333/PN.50150263.V1.1) and are available to any researcher upon application and approval. Under the MDIS terms of use, the authors are not permitted to redistribute the microdata or respondent-level files derived from them. To enable full replication, the Supplementary Materials provide a codebook specifying the construction of every analytic variable from the source items, together with the complete Stata and R analysis code.

## Supplementary Materials

The following supporting information can be downloaded at: https://www.mdpi.com/article/10.3390/bs16101742/s1, Table S1: Rotated component loadings for the 13 digital device items (principal component analysis, Varimax rotation with Kaiser normalization; Stata/SE 16.1); Table S2: Sensitivity analysis based on tetrachoric correlations; Table S3: Incremental variance explained by the interaction blocks; Table S4: Sensitivity analysis incorporating survey weights; Table S5: Categorical confirmatory factor analysis (WLSMV, N = 9951); Table S6: Extended covariate sensitivity analysis (N = 9837); Table S7: Weight- and cluster-adjusted estimation (survey weight + enumeration-district clustering); Table S8: Random split-sample check of the two-component structure; Table S9: Associations using the 10-item summed score and tests of domain coefficient equality.
