# Supplementary material for: Disaggregating Digital Competence: Social Activity Participation and Depressive Symptoms Among Korean Older Adults
Source: Behav Sci (Basel). 2026 Sep 24;16(10):1742. doi: 10.3390/bs16101742 (PMC13702013; doi:10.3390/bs16101742)
Supplement: Supplementary file 1 [file behavsci-16-01742-s001.zip › behavsci-4510390-supplementary.pdf]

# Supplementary Material

Disaggregating Digital Competence: Social Activity Participation and Depressive Symptoms among Korean Older Adults

Data: 2023 National Survey of Older Koreans, analytic sample N = 9,951. Primary analyses were conducted in Stata/SE 16.1; confirmatory factor analyses and the tetrachoric parallel analysis were estimated in R 4.6.1 (lavaan 0.7-2, psych); selected estimates were independently cross-checked in Python 3.12. Bootstrap analyses used 5,000 resamples with percentile confidence intervals and random seed 20260718.

## Table S1. Rotated component loadings for the 13 digital device items (principal component analysis, Varimax rotation with Kaiser normalization; Stata/SE 16.1)

| Item | Component 1 (life-service) | Component 2 (communication-media) |
|---|---|---|
| E-commerce | .796 | .113 |
| Financial transactions | .758 | .247 |
| Application installation | .805 | .140 |
| Kiosk ordering | .718 | .222 |
| Receiving messages | −.018 | .771 |
| Sending messages | .075 | .844 |
| Taking photographs | .335 | .759 |
| Searching for information | .357 | .752 |
| Video calling | .319 | .673 |
| Watching videos | .349 | .667 |
| Listening to music (excluded) | .523 | .457 |
| Playing games (excluded) | .522 | .267 |
| Social networking services (excluded) | .637 | .114 |

Note. Eigenvalues of the first four components: 5.802, 1.768, 0.860, 0.728. Loadings of retained items on their own component appear in Table 2 of the main text. Listening to music cross-loaded on both components; playing games showed only a modest primary loading; social networking service use loaded primarily on the life-service component and was excluded on conceptual grounds. An oblique Promax rotation (R psych, principal components, m = 4; script parallel_tet.R) reproduced the same item assignment (communication-media items .65–.94; life-service items .74–.87; component correlation = .55), indicating that the assignment is not an artifact of the orthogonality constraint.

## Table S2. Sensitivity analysis based on tetrachoric correlations

Because the 13 indicators are dichotomous, the correlation structure was re-estimated using tetrachoric correlations (R psych). The eigenvalues of the tetrachoric matrix were 9.911, 0.771, 0.499, 0.408, 0.324, 0.301, 0.220, 0.166, 0.128, 0.112, 0.099, 0.055, and 0.007; only the first exceeded 1. A parallel analysis in which both the observed and the simulated matrices were tetrachoric-based (psych::fa.parallel, cor = "tet", 100 iterations, seed 20260718) recommended one component under the principal-component criterion; the common-factor criterion suggested five factors. Neither criterion supported exactly two factors, indicating a strong general competence factor.

When two factors were nonetheless extracted from the tetrachoric matrix (minimum-residual estimation, Varimax rotation), the item grouping of the main analysis was reproduced: the six communication-media items loaded .760–.830 on one factor and the four life-service items loaded .714–.837 on the other, with substantial cross-loadings (≥ .37) consistent with a strong general factor.

| **Item** | **Factor 1** | **Factor 2** |
|---|---|---|
| E-commerce | .426 | .837 |
| Financial transactions | .480 | .794 |
| Application installation | .446 | .830 |
| Kiosk ordering | .499 | .714 |
| Receiving messages | .830 | .424 |
| Sending messages | .823 | .491 |
| Taking photographs | .782 | .527 |
| Searching for information | .782 | .541 |
| Video calling | .765 | .367 |
| Watching videos | .760 | .400 |
| Listening to music (excluded) | .701 | .453 |
| Playing games (excluded) | .618 | .425 |
| Social networking services (excluded) | .504 | .607 |

## Table S3. Incremental variance explained by the interaction blocks

Each row adds the two interaction terms (life-service × moderator, communication-media × moderator) to the direct-path model including the mediator and all covariates, i.e., the same specification as the moderation analyses reported in the main text.

| **Moderator** | **$R^2$ base model** | **$R^2$ with interactions** | **$\Delta R^2$** | **Cohen's $f^2$** |
|---|---|---|---|---|
| Age (old-old) | .10728 | .10739 | .00011 | .00012 |
| Education | .10728 | .10837 | .00109 | .00122 |
| Living alone | .10728 | .10752 | .00025 | .00027 |

Note. Cohen’s (1988) benchmarks: $f^2$ = .02 small, .15 medium, .35 large. All interaction effects fall below the conventional threshold for a small effect and should be interpreted accordingly.

## Table S4. Sensitivity analysis incorporating survey weights

The direct-effect model (Model 3 specification) was re-estimated by weighted least squares using the standardized post-stratification weight provided with the survey, with heteroskedasticity-robust (HC1) standard errors.

| Predictor | B (unweighted) | p | B (weighted) | p |
|---|---|---|---|---|
| Female | −0.134 | .045 | −0.095 | .215 |
| Old-old (75+) | +0.067 | .369 | +0.240 | .012 |
| Education (3-category) | −0.256 | < .001 | −0.148 | .035 |
| Living alone | +0.498 | < .001 | +0.527 | < .001 |
| Life-service competence | −0.179 | < .001 | −0.244 | < .001 |
| Communication-media competence | −0.158 | < .001 | −0.112 | < .001 |
| Social activity participation | −0.870 | < .001 | −1.008 | < .001 |

Note. The focal associations (both digital competence dimensions and social activity participation) retained their sign and significance under weighting. Some covariate coefficients shifted (sex, age group, education), so weighted and unweighted estimates are not interchangeable for the covariates.

## Table S5. Categorical confirmatory factor analysis (WLSMV, N = 9,951)

A one-factor model and a correlated two-factor model were estimated in R (lavaan) with WLSMV estimation, treating the 10 retained items as ordered-categorical indicators.

| Model | Scaled $\chi^2$ (df) | CFI | TLI | RMSEA [90% CI] | SRMR |
|---|---|---|---|---|---|
| One-factor | 1176.20 (35) | .996 | .995 | .057 [.054, .060] | .069 |
| Two-factor (correlated) | 520.15 (34) | .998 | .998 | .038 [.035, .041] | .034 |

Note. Scaled chi-square difference test (Satorra, 2000): $\Delta\chi^2(1) = 303.68$, $p < .001$, favoring the two-factor model. Standardized loadings (two-factor model): life-service .895–.940 (e-commerce .922, financial transactions .940, application installation .929, kiosk ordering .895); communication-media .839–.986 (receiving messages .986, sending messages .973, photographs .935, information search .953, video calling .839, watching videos .850). Factor correlation = .878 (SE = .007).

## Table S6. Extended covariate sensitivity analysis (N = 9,837)

Models were re-estimated adding marital status (spouse present), employment, self-rated health, number of chronic conditions, ADL limitations, IADL limitations, cognitive function score, and fall experience to the demographic covariates. Bootstrap: 5,000 resamples, seed 20260718.

| Quantity | Primary model B (p) | Extended model B (p) |
|---|---|---|
| Life-service: total association | −0.251 (< .001) | −0.112 (< .001) |
| Life-service: direct association | −0.179 (< .001) | −0.081 (.004) |
| Communication-media: total association | −0.231 (< .001) | −0.017 (.340) |
| Communication-media: direct association | −0.158 (< .001) | +0.012 (.507) |
| Social activity participation | −0.870 (< .001) | −0.411 (< .001) |
| Indirect: life-service [95% CI] | −.072 [−.086, −.059] | −.031 [−.040, −.022] |
| Indirect: communication-media [95% CI] | −.073 [−.085, −.063] | −.028 [−.036, −.021] |
| Communication-media × education interaction | +0.085 (.001) | +0.006 (.780) |

Note. Both indirect associations and the life-service direct association are robust to extended adjustment; the communication-media direct association and its moderation by education are not. Several extended covariates (self-rated health, functional limitations, cognitive function) are plausibly on the causal pathway or share causes with digital competence, so the extended models may over-control; the demographic-adjusted models are treated as primary.

## Table S7. Weight- and cluster-adjusted estimation (survey weight + enumeration-district clustering)

Models were re-estimated by weighted least squares using the standardized post-stratification weight, with cluster-robust (CR1) standard errors across 977 enumeration districts. Indirect associations were bootstrapped by resampling enumeration districts (5,000 resamples, seed 20260718).

| Quantity | Estimate | p / 95% CI |
|---|---|---|
| Model 1 (social activity): life-service | +0.088 | < .001 |
| Model 1 (social activity): communication-media | +0.082 | < .001 |
| Model 2 (total): life-service | −0.333 | < .001 |
| Model 2 (total): communication-media | −0.195 | < .001 |
| Model 3 (direct): life-service | −0.244 | < .001 |
| Model 3 (direct): communication-media | −0.112 | < .001 |
| Model 3 (direct): social activity participation | −1.008 | < .001 |
| Indirect: life-service [95% CI] | −.089 [−.113, −.065] | excludes 0 |
| Indirect: communication-media [95% CI] | −.083 [−.101, −.067] | excludes 0 |

Note. All focal associations retain their sign and significance under weight-and-cluster adjustment.

## Table S8. Random split-sample check of the two-component structure

The analytic sample was randomly split in half in R with set.seed(20260718). Principal component analysis with Varimax rotation was estimated in the exploratory half, and the confirmatory one- versus two-factor comparison (WLSMV) was estimated in the other half. The split and both analyses are reproduced by cfa_wlsmv.R, Part 2.

| Analysis | Result | Model comparison |
|---|---|---|
| PCA half (n = 4,975) | Eigenvalues 5.827 / 1.760; identical item assignment to the full-sample solution | |
| CFA half (n = 4,976): one-factor model | CFI = .995; TLI = .994; RMSEA = .062; SRMR = .068 | |
| CFA half (n = 4,976): two-factor model | CFI = .998; TLI = .998; RMSEA = .040; SRMR = .034; factor correlation = .87 | Scaled $\Delta\chi^2(1) = 194.50$, $p < .001$ |

Note. The two-factor model was again preferred in the confirmatory half, with the same high factor correlation observed in the full sample.

## Table S9. Associations using the 10-item summed score and tests of domain coefficient equality

The summed score contains the same ten items as the two domain scores (range 0–10). Panel A reports the focal regression coefficients; Panel B reports indirect associations; Panel C tests equality of the unstandardized domain coefficients.

Specifications: A, primary adjustment, unweighted (N = 9,951); B, extended adjustment, unweighted (N = 9,837); C, primary adjustment, weighted and clustered (N = 9,951); D, extended adjustment, weighted and clustered (N = 9,837).

### Panel A. Summed-score regression models

| ID | Association | B | SE | 95% CI | p |
|---|---|---|---|---|---|
| A | Score → social activity (a) | .084 | .002 | [.079, .088] | < .001 |
| A | Score → depression, total (c) | −.238 | .014 | [−.264, −.211] | < .001 |
| A | Score → depression, direct (c′) | −.165 | .014 | [−.193, −.137] | < .001 |
| A | Social activity → depression (b) | −.870 | .059 | [−.986, −.754] | < .001 |
| B | Score → social activity (a) | .071 | .002 | [.066, .075] | < .001 |
| B | Score → depression, total (c) | −.048 | .012 | [−.072, −.024] | < .001 |
| B | Score → depression, direct (c′) | −.019 | .013 | [−.044, .006] | .142 |
| B | Social activity → depression (b) | −.412 | .052 | [−.515, −.309] | < .001 |
| C | Score → social activity (a) | .084 | .004 | [.077, .092] | < .001 |
| C | Score → depression, total (c) | −.242 | .020 | [−.282, −.202] | < .001 |
| C | Score → depression, direct (c′) | −.157 | .021 | [−.199, −.115] | < .001 |
| C | Social activity → depression (b) | −1.010 | .092 | [−1.190, −.829] | < .001 |
| D | Score → social activity (a) | .071 | .004 | [.064, .079] | < .001 |

| ID | Association | B | SE | 95% CI | p |
|---|---|---|---|---|---|
| D | Score → depression, total (c) | −.054 | .019 | [−.092, −.017] | .005 |
| D | Score → depression, direct (c′) | −.015 | .020 | [−.054, .024] | .451 |
| D | Social activity → depression (b) | −.550 | .082 | [−.711, −.388] | < .001 |

Note. All coefficients are unstandardized. The a model predicts social activity; the c model predicts depressive symptoms without the mediator; the c′ and b coefficients come from the same model including social activity. Covariate coefficients and intercepts are omitted. Confidence intervals in Panel A are model-based.

## Table S9. Continued

### Panel B. Indirect associations through social activity participation

| ID | N | Indirect association (a × b) | Bootstrap SE | Percentile 95% CI |
|---|---|---|---|---|
| A | 9,951 | −.073 | .005 | [−.083, −.063] |
| B | 9,837 | −.029 | .004 | [−.037, −.022] |
| C | 9,951 | −.085 | .008 | [−.101, −.070] |
| D | 9,837 | −.039 | .006 | [−.051, −.028] |

Note. Point estimates are products of coefficients fitted to the original sample, not averages across bootstrap resamples. Percentile intervals use 5,000 resamples (NumPy default_rng seed 20260718): individuals for A and B; enumeration districts, retaining survey weights within each sampled district, for C and D. All four intervals exclude zero.

### Panel C. Equality tests in the corresponding disaggregated models

| ID | a path equality p | Total path equality p | LS direct B | CM direct B | Direct path equality p |
|---|---|---|---|---|---|
| A | .810 | .650 | −.179 | −.158 | .621 |
| B | .411 | .010 | −.081 | .012 | .012 |
| C | .643 | .015 | −.244 | −.112 | .018 |
| D | .327 | < .001 | −.135 | .047 | < .001 |

Note. LS = life-service competence; CM = everyday communication and media-use competence. Each equality test evaluates the null hypothesis that the two domain coefficients are equal in the specified path model. Because the summed score is LS + CM, it imposes a common per-item coefficient. Failure to reject this restriction is not evidence of equivalence, and these tests do not compare fully standardized indirect associations.

Adjustment and estimation. Primary covariates are sex, age group, education (three-category ordinal coding), and living alone. Extended models additionally include spouse present, employment, self-rated health, number of chronic conditions, ADL limitations, IADL limitations, cognitive function, and fall experience. The extended sample excludes 114 respondents with missing IADL information. A and B use ordinary least squares with conventional standard errors and t inference. C and D use weighted least squares with the survey's standardized post-stratification weight and CR1 cluster-robust standard errors across 977 enumeration districts; p values and model-based intervals use the normal reference distribution. All estimates describe cross-sectional associations.

## References

Cohen, J. (1988). *Statistical power analysis for the behavioral sciences* (2nd ed.). Lawrence Erlbaum Associates.

Satorra, A. (2000). Scaled and adjusted restricted tests in multi-sample analysis of moment structures. In R. D. H. Heijmans, D. S. G. Pollock, & A. Satorra (Eds.), *Innovations in multivariate statistical analysis: A festschrift for Heinz Neudecker* (pp. 233–247). Kluwer Academic.
